# Development and Application of a Patient Group Engagement Prioritization Tool for Use in Medical Product Development

**DOI:** 10.1007/s43441-020-00217-0

**Published:** 2020-09-29

**Authors:** Brian Perry, Carrie Dombeck, Jaye Bea Smalley, Bennett Levitan, David Leventhal, Bray Patrick-Lake, Linda Brennan, Kevin McKenna, Zachary Hallinan, Amy Corneli

**Affiliations:** 1 Clinical Trials Transformation Initiative, Duke University, Durham, NC USA; 2 Department of Population Health Sciences, Duke University, 215 Morris Street, Suite 210, Durham, NC 27701 USA; 3 Patient Advocate, New York, NY USA; 4 Janssen R&D LLC, Titusville, NJ USA; 5 Pfizer, Inc, Groton, CT USA; 6 Duke Clinical Research Institute, Durham, NC USA; 7 Cystic Fibrosis Foundation, Washington, DC USA

**Keywords:** Patient engagement, Stakeholder engagement, Patient group engagement, Prioritization tool, Patient engagement activities

## Abstract

**Introduction:**

Patient group engagement is increasingly used to inform the design, conduct, and dissemination of clinical trials and other medical research activities. However, the priorities of industry sponsors and patient groups differ, and there is currently no framework to help these groups identify mutually beneficial engagement activities.

**Methods:**

We conducted 28 qualitative, semi-structured interviews with representatives from research sponsor organizations (*n* = 14) and patient groups (*n* = 14) to determine: (1) how representatives define benefits and investments of patient group engagement in medical product development, and (2) to refine a list of 31 predefined patient group engagement activities.

**Results:**

Patient group and sponsor representatives described similar benefits: engagement activities can enhance the quality and efficiency of clinical trials by improving patient recruitment and retention, reduce costs, and help trials meet expectations of regulators and payers. All representatives indicated that investments include both dedicated staff time and expertise, and financial resources. Factors to consider when evaluating benefits and investments were also identified as were suggestions for clarifying the list of engagement activities.

**Discussion:**

Using these findings, we refined the 31 engagement activities to 24 unique activities across the medical product development lifecycle. We also developed a web-based prioritization tool (https://prioritizationtool.ctti-clinicaltrials.org/) to help clinical research sponsors and patient groups identify high-priority engagement activities. Use of this tool can help sponsors and patient groups identify the engagement activities that they believe will provide the most benefit for the least investment and may lead to more meaningful and mutually beneficial partnerships in medical product development.

**Electronic supplementary material:**

The online version of this article (10.1007/s43441-020-00217-0) contains supplementary material, which is available to authorized users.

## Introduction

Over the past decade, patients have collaborated with researchers, funders, academia, and sponsors to inform research priorities, funding decisions, health services research, the selection of outcomes, clinical trial protocol designs, and recruitment and retention [1, 2]. Because patient group engagement has significant potential to improve the clinical trial enterprise, the Clinical Trials Transformation Initiative (CTTI)—a public–private partnership co-founded by Duke University and the US Food and Drug Administration (FDA) whose members include representatives from across the clinical trials ecosystem—developed the Patient Groups and Clinical Trials project in 2013 to foster this collaboration. Initially the project sought to better understand stakeholders’ perceptions regarding the importance and value of engaging patient groups and the various clinical trial services that patient groups provide. This led to the development of recommendations, best practices, and a list of specific activities for engaging patient groups throughout the clinical trial process [3]. To further advance mutually beneficial patient group engagement, the project developed a financial model to better articulate the impact that patient engagement may have on key business drivers and to demonstrate that return on investment should support broader adoption [4].

This work, along with emerging best practice resources on patient engagement in clinical trials, such as those from the Patient-Focused Medicines Development (PFMD) [5] and FasterCures [6], is helping to accelerate patient group engagement. While there is still much to understand, engaging patient groups in clinical trials is also gaining broader acceptance: for example, the Patient-Centered Outcomes Research Institute (PCORI) requires patient engagement in any of their funded clinical trials [7]; the National Academy of Medicine (NAM) recommends including patients as partners in research [8], and the US FDA has acknowledged the importance of patient involvement through a range of initiatives and guidance documents [9–12].

However, despite resources to help stakeholders understand the breadth of potential patient engagement activities and promising practices [13], there is no widely used framework or method to facilitate identifying fit-for-purpose activities that are mutually beneficial for the sponsor and for the patient group or the patient community they represent. To support this need, CTTI has developed a framework and a prioritization tool to aid both sponsors and patient groups in determining, from their perspective, (1) the benefit that patient group engagement can bring to their organizations and the clinical trial process, (2) the investment that such engagement would require, and (3) those engagements that are of highest priority to each organization. The tool supports users—both patient groups and sponsors—in identifying relevant engagement opportunities for a specific study, subjectively assessing the benefits and investments of each (low, moderate, high), and visualizing and discussing the output together as partners.

This manuscript describes the process CTTI used to gather evidence to develop the tool, describes the tool itself, and describes how the tool can be used by sponsors and patient groups to guide decisions on priority patient group engagement activities.

## Methods

### Evidence Gathering

Working from the CTTI Patient Group Organizational Expertise and Assets evaluation tool, we developed a list of 31 patient group engagement activities in medical product development [14]. We conducted 28 qualitative, semi-structured interviews (SSIs) with representatives involved in engaging patients in medical product development from research sponsor organizations (*n* = 14) and patient groups (*n* = 14), from January 26, 2017, to April 18, 2017 (Table 1). Representatives were purposively selected [15] based on their knowledge of the types of patient group engagement activities their organization has participated in and whether their organization is actively engaged in medical product development. In addition, we purposively recruited representatives from organizations of varying sizes (e.g., based on annual budget) and organizations involved in medical product development across the clinical trial continuum (i.e., pre-discovery through post-approval). Representatives were asked to review each of the 31 CTTI patient group engagement activities [14] and consider the relative benefit of each activity. They were instructed to categorize each engagement activity as either providing a *high*, *moderate*, *low,* or *no benefit* to their constituents or company using an interactive online pile sorting platform created for this study. They were then asked to describe their rationale for their ratings. Following the “benefits” questions, representatives individually reviewed the same 31 engagement activities again, considering whether the activity would require a relatively *high*, *moderate*, *low*, or *no investment* to perform. After classifying all of the activities, participants were asked to explain their rationale for determining the investment category for the activities. We also asked the participants if any of the 31 patient group engagement activities were unclear and if so, how to refine the description of the activity.

**Table 1:** Demographics.

Patient Groups	*n* (%)	Industry Sponsors	*n* (%)
Size of Company			
(Approximate Annual Budget)		(Approximate Market Cap)	
Less Than $500,000	1 (7)	Under $300 Million	1 (7)
$500,000 to $999,999	1 (7)	$300 Million to Under $2 Billion	2 (14)
$1,000,000 to $4,999,999	4 (29)	Between $2 Billion and $10 Billion	3 (21)
$5,000,000 to $9,999,999	3 (21)	Over $10 Billion	8 (57)
$10,000,000 or greater	5 (36)		
Disease or Health Condition Focus (Select All that Apply)			
Rare Diseases	7 (50)	All/Nonspecific	9 (64)
Rare Genetic Disorders	5 (36)	Nervous System Disorders/Mental Health	2 (14)
Rare Cancers	2 (14)	Rare Diseases	2 (14)
Common Diseases	7 (50)	Cancers	1 (7)
General Cancers	2 (14)		
Neurological Diseases	2 (14)		
Autoimmune Diseases	2 (14)		
Respiratory/Pulmonary Diseases	1 (7)		
Years of Organization has been Engaged in Medical Product Development			
Less than One Year	0 (0)	Less than One Year	1 (7)
1 to 2 Years	1 (7)	1 to 2 Years	1 (7)
3 to 4 Years	1 (7)	3 to 4 Years	5 (36)
5 to 10 Years	0 (0)	5 to 10 Years	3 (21)
More than 10 Years	11 (79)	More than 10 Years	1 (7)
Not Sure	1 (7)	Not Sure	2 (14)
No Response	0 (0)	No Response	1 (7)
Engagement in Phases of Medical Product Development (Select All that Apply)			
Pre-Discovery	13 (93)	Pre-Discovery	4 (29)
Preclinical	14 (100)	Preclinical	7 (50)
Phase 1, Phase 2, and/or Phase 3 Trials	14 (100)	Phase 1, Phase 2, and/or Phase 3 Trials	14 (100)
FDA Review & Approval	7 (50)	FDA Review & Approval	7 (50)
Post-Approval	6 (43)	Post-Approval	8 (57)

All interviews were audio recorded and transcribed verbatim. We used applied thematic analysis to analyze the data [16]. NVivo 11 software was used to organize and code the transcripts [17]. Three analysts initially coded each of the transcripts using an apriori coding structure based on questions in the interview guide. Inter-coder reliability was assessed on 10% of the transcripts. Any discrepancies in how these codes were applied were resolved through group discussion and edits were made to the codebook to aid in future application of the codes. Next, all coded text related to the initial coding structure was reviewed for information that revealed representatives’ beliefs about the benefits of and investments required for engaging patients in medical product development and also to refine the 31 activities. This information was coded and thematically organized by two trained qualitative analysts using a process of constantly comparing new information to information previously identified and coded. The data organized within the emergent thematic groups were verified by a third analyst. Finally, coding frequencies and matrices were reviewed to identify themes that were common across patient group and sponsor representatives, as well as those that were differentially expressed by certain groups, or possibly idiosyncratic. Themes were described in analytical memos, which were used to present the results below.

## Results

### Benefits of Patient Group Engagement

Patient group and sponsor representatives described similar potential benefits of patient group engagement (Table 2). Both groups suggested that patient group engagement can enhance the quality and efficiency of clinical trials by improving patient recruitment and retention, by reducing costs, and by making trials more able to meet expectations of regulators and payers. Other benefits suggested by the representatives include reducing the burden of participation by optimizing trial design and conduct, and amplifying the patient voice in medical product development, thereby improving the product’s ability to more directly address patient needs. In addition, sponsor representatives indicated that patient group engagement in clinical research motivates research staff, patient groups, and ultimately trial participants (if patient groups remain engaged throughout the trial period), which helps ensure that the trial is conducted well. Respondents also noted that patient group involvement in clinical research can strengthen grassroots advocacy of clinical trials and enhance the reputation of the sponsor, trial, and product in the public sphere.

**Table 2:** Quotes Regarding Benefits to Patient Engagement in Medical Product Development.

Benefit	Quotes from Patient Group Representatives	Quotes from Industry Sponsor Representatives
Improving the Quality and Efficiency of Clinical Trials	We want to make sure that industry understands that we have a registry in place and that we can enroll trials quickly. And that if they come into our community, we're going to help them, however we can, through the clinical trial process. So I think ultimately our goal is to bring new safe, effective products to our patient community. And the more we can bridge some gaps and fill in where our expertise is—we want to make it easy for the scientists, we want to make it easy for the clinicians, we want to make it easy for industry, and we want to make it easy for FDA	[Patient engagement] helps get medicines that are meaningful to patients to them faster If you're designing, say, clinical trials or any materials that have patient input, then it will be more attractive to patients as a whole. So a clinical trial will be potentially simpler for patients to participate, and then we are getting benefit from improved recruitment or enrollment in a clinical trial that gets the medicine to market faster for patients
Amplifying the Patient Voice and Address Patient Needs	We always [keep] the patient in mind. We were founded by patients. Our money comes from patients and their families. So we want to make sure that it’s really addressing something that matters to patients	We’ve certainly worked to get the patient voice into advisory committees, even how we really think about our submission documents and what kind of strategy…what gets focused on, what are the key components that come out of our submission documents in terms of what we emphasize. The way the patient views that benefit/risk equation might be different than maybe how a regulator might see it
Motivating Patients and Researchers to Get More Involved	The benefit is that the more motivated our organization becomes with our participants, the more motivated the researchers become in their work	For our own colleagues, when they’ve engaged with patients they feel more motivated, they feel more inspired. We believe [the effect is positive] in terms of retention of colleagues, productivity of colleagues and just the general effect on the workforce
Strengthen Grassroots Advocacy of Clinical Trials	I think that what we've been able to do is mobilize a grassroots effort over time that is sophisticated and knowledgeable about drug development. We've empowered tiny family foundations and we gave them the tools to advocate We bring our community volunteers from across the country to advocate for a DOD appropriation for cancer research annually That benefits our community by making sure that the FDA understands the unmet needs in our community and is kind of primed for understanding what would be the main benefits of a drug	Deeper relationships with the advocacy community helps individuals feel like they've been a part of the development of a new medicine, and are therefore willing and able to champion it to ensure that it gets to people who need it Better relationship with the community has far-reaching impacts that you wouldn't necessarily anticipate. It's not just about learning how we can make existing medications better, but also potentially sparking ideas for unmet needs we weren't aware of, helping to build greater bridges between the community and how the industry is viewed as a whole I think it really makes a difference for how the patient community views the pharmaceutical industry … and that we truly are partners in disease management and not what tends to be recorded on the front page of the Wall Street Journal
Enhancing the Reputation of the Sponsor, Trial	No comments	I think we think that engaging with patient advocacy groups brings a positive reputational benefit to [company’s name]. We feel it’s the right thing to do, and by putting our money where our mouth is and actually doing it, we do feel it probably helps us with reputation

### Considerations Made When Evaluating Benefits

Representatives reported that the level of benefit offered by each of the 31 CTTI patient group engagement activities was determined by subjectively assessing one or more of the following factors:

- The extent of the effect of the activity on the patient population or organization. For example, some patient group engagement activities could affect a large segment of the patient population or could affect several future trials.
- The necessity of patient group involvement to conduct the activity.
- The necessity of the activity to advance medical product development.
- The ease of accomplishing the activity in the short term.
- Reputational benefits gained by conducting the activity. For example, some patient group engagement activities might be perceived by patients and other stakeholders in the community as “the right thing to do.”

### Investments in Patient Group Engagement

All representatives indicated that the investments required for successful patient group engagement include dedicated staff time and expertise, as well as financial resources, all of which can be impacted by the scope and longevity of the specific engagement activity (Table 3). Investments also could include the creation of new infrastructure, processes, and organizational policies to facilitate the activity. Representatives noted that some engagement activities may require additional time, effort, or burden placed directly on patients, which may be a cost that some groups are unable or unwilling to afford. Finally, a patient group representative reported that organizations may need to consider whether or not engaging in a particular activity, or associating themselves with a particular research partner, will cost them their reputation or ethical principles.

**Table 3:** Quotes Regarding Investments to Patient Engagement in Medical Product Development.

Investments	Quotes from Patient Group Representatives	Quotes from Industry Sponsor Representatives
Dedicated Staff Time and Expertise	Our staff is small, so it’s like, our knuckles are sbleeding right now doing the work that we're doing. We don't have a lot of staff to accomplish these jobs, so everything [involves] quite a bit of investment for us. It's time, power and money that I don't have to hire somebody else and get the job done	So we actually have a whole new team. So I am focused on patient engagement, but there’s actually also other people on the—I have peers that are focused on like investigator and site engagement as well. So it’s a really kind of dedicated effort to think about from not just the patient perspective but also from the site and investigator perspective, how do we just do a better job? My role also came out of just a company wide recognition that we need to be thinking about patients differently. And not just sort of the end user Putting a team in place that can assist the clinical team with establishing those networks and opportunities for actually engaging with the patients or the patient groups or that list of different ways or different populations that you might work with to reach the patient I guess a big investment is just dedicated staff to run the activities, to maintain the partnerships with the external groups
Financial Resources	The big thing is money, and to raise more money. Our annual budget, with the creation of the registry and the care center network, has gone up substantially. And the more patients you enroll, and the more centers that you engage, the more money it costs. I'm focusing a lot of my energies right now on development and fund raising	We invest funds; so we have a sponsorship and a grants program that invest dollars directly to do these programs. We invest direct funds through partnership programs Well investments in terms of finances for sure. The FDA suggested that we hold a patient-focused drug development meeting. So we had to give a very sizeable grant to an advocacy group
New Infrastructure, Processes, and Organizational Policies	For other diseases, many already had experience in this arena, but we didn’t, and so we were starting from scratch. Because we created this from the ground up, it’s really building it from nothing. It’s an enormous amount of work, effort, learning, getting up to speed, meetings, funding, whatever it may be. Starting from scratch takes a lot of investment	The key investment is internal education, and for us at [company], that's really been a cultural change. Within the organization, to really put the patient at the center of what we do has been a significant change in how [company] operates and where we put our focus. That's required a resource investment on the part of [company], both by bringing on additional personnel or realigning personnel within the organization who will dedicate their full effort Although very cool and important, a lot of these [activities] seemed like a very heavy lift from a resource perspective in terms of getting kind of agreement across the organization so that we’d be able to do these things
Time, Effort and Burden Placed Directly on Patients	There are logistic investments in some of these things, and you’re working with people with [name of disease], it’s not easy for them to travel. You have to consider that aspect of it	No comments
Reputation or Ethical Principles	We’ve tried not to sell our soul, and that’s a very important issue for us. I’ll give you an example. Years ago, we brought our board together, and the issue was a clinical trial for a drug… and the clinical trial might have placebo. We met for a day. I remember it was a very unusual board meeting. We had board members in tears as they agonized over the following question. We had come to the conclusion that we felt that the placebo was not necessary, and that we thought that the placebo was not very good science. We didn’t want to know whether their drug was better than nothing. We also felt that we did not want to put patients who have [name of condition] in that kind of a situation. The discussion was if we oppose this clinical trial, it’s going to cost us a relationship with [the sponsor]; including a financial relationship in terms of further support. If we don’t, it costs us our soul. We voted for our soul. In fact, it did cost us. We adopted a principle that, so far, we’ve been able to follow. The principle was: does it meet the litmus test of what one needs to do to keep patients alive and well? … If it doesn’t meet that test, we’ll take the consequences of it	No comments

### Considerations Made When Evaluating Investments

Representatives indicated that the level of investment for each engagement activity was determined by subjectively assessing one or more of the following factors:

- The amount of financial resources needed to conduct the activity. For example, some patient group engagement activities might be longer-term and require continual financial investment.
- The level of staff time and expertise required across the lifespan of the activity.
- The amount of organizational commitment needed, given existing infrastructure. For example, some patient group engagement activities might demand a great deal of commitment from the organization to establish necessary infrastructure and processes.
- The amount of direct patient involvement and potential patient burden. For example, some patient group engagement activities might necessitate interacting directly with patient populations and require a great deal of patients' time and effort.
- Reputational risks posed by engaging in the activity. For example, some patient group engagement activities might pose a potentially serious risk to the reputation of the sponsor or patient group if not done well or if the partnership is perceived to violate the ethos of the organization.

### Modifications to the 31 Patient Group Engagement Activities

**Figure 1:**
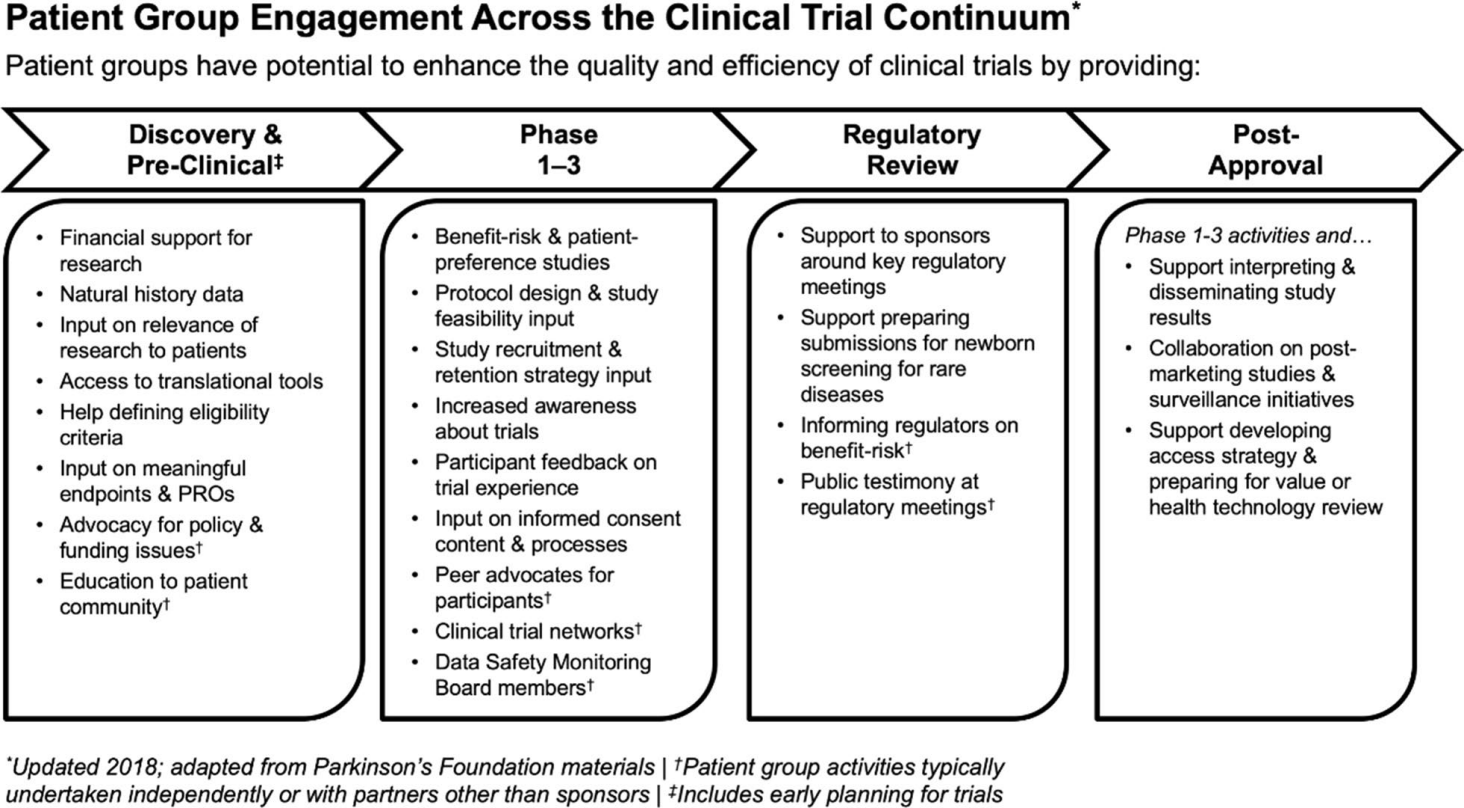
Refined List of Patient Group Engagement Activities.

Representatives also suggested ways to refine the original list of 31 patient group engagement activities, such as clarifying any unclear descriptions of engagement methods, combining methods that were similar, and identifying any other engagement methods they felt were missing from the original list. Suggested modifications were compiled and used to condense the list of patient group engagement activities to 24 unique activities across the medical product development lifecycle (Fig. 1; see supplemental material for further description of each engagement activity).

## Discussion

Although best practices and research for assessing patient group engagement are still evolving, this type of collaboration is recognized as having the potential to significantly improve the clinical trial enterprise [2, 18]. Ensuring that collaboration is focused on areas where the greatest benefit can be achieved for everyone involved, given limited resources, is an important step in the development of strong partnerships to improve the relevance of information gathered from clinical trials.

### CTTI Prioritization Tool for Sponsors and Patient Groups

We used the findings in these interviews to develop a web-based tool to help clinical research sponsors and patient groups, both individually and jointly, identify high-priority patient group engagement activities that will be most relevant to their clinical research interests and needs.

The resulting "prioritization tool" supports users in identifying engagement activities that are most relevant to their situation (e.g., a particular clinical trial, or a collaboration across a development program), and provides a framework for transparent and intentional decision-making. The tool is available on the CTTI website: https://prioritizationtool.ctti-clinicaltrials.org/.

The tool seeks to assist users in identifying:

1. Relevant engagement activities that would be of most value (high benefit and low investment) to pursue on their own or in partnership
2. Engagement activities that would be beneficial for their constituents but that may be too costly to invest in (high benefit and high investment activities)
3. Engagement activities that provide little direct benefit or cost to their constituents (low benefit and low investment activities) but could potentially be valuable to other strategic research partners
4. Engagement activities that are unlikely to be worth pursuing (low benefit, high investment activities).

By identifying these specific engagement activities, the user will be able to better choose which activities they would seek to gain in a new research partnership, as well as what they may have to offer potential partners. Then the partners can allocate resources to those projects that are of the most value jointly to both organizations.

### Application of the Prioritization Tool

**Figure 2:**
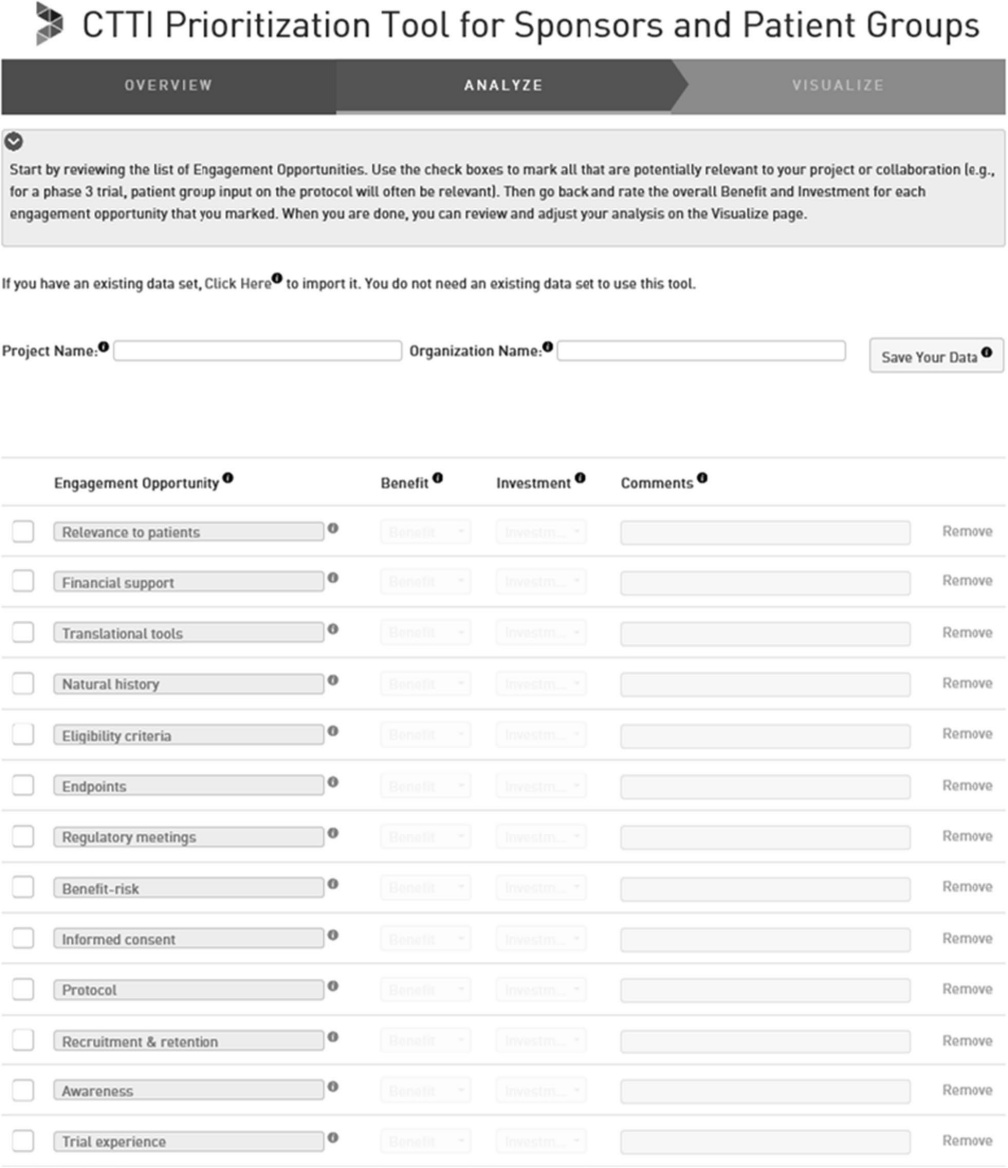
Step 1—Identify Fit-for-Purpose Patient Engagement Activities.

The tool walks users through completing the following 3-step decision-making process:

Step one (Fig. 2) involves patient groups and research sponsors—either working together or independently—identifying relevant engagement activities. Users of the tool are provided examples of each of the 24 patient engagement activities identified by CTTI (Fig. 1) and can also choose to add their own fit-for-purpose activities.

**Figure 3:**
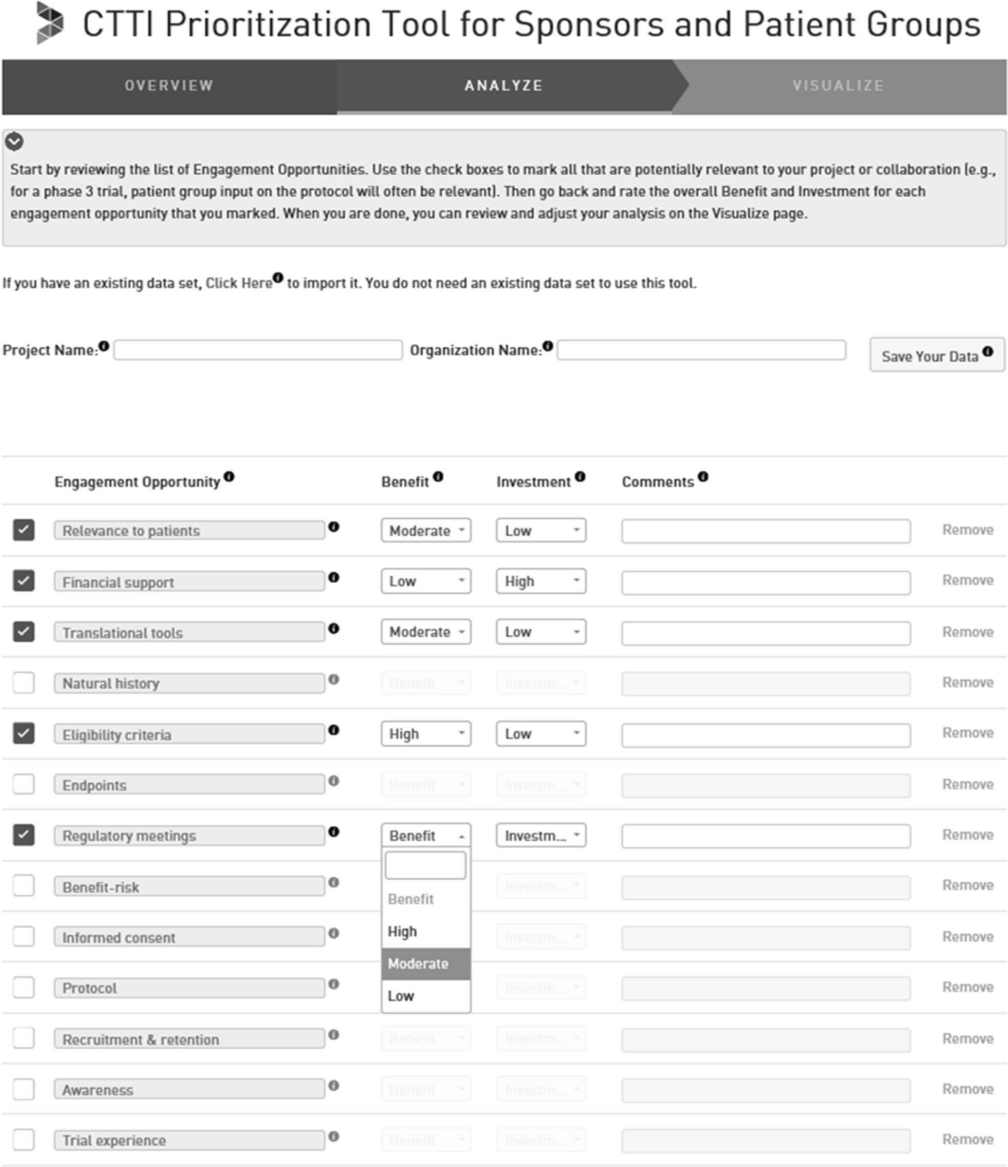
Step 2—Evaluate Level of Benefit and Investment of Each Patient Engagement Activity.

Step two (Fig. 3) involves evaluating the relative benefits and investments associated with each activity that was identified as relevant in step one. For each engagement activity, users are instructed to assess the expected level of benefit the activities will provide to their organization or constituents and the expected level of investment it would take their organization to accomplish the activity. To help evaluate the potential level of benefit offered and investment required by a particular engagement activity, the tool suggests that users consider the factors described above. These assessments are intentionally subjective, as detailed financial or strategic modeling is often unrealistic for projects at this stage. At this time users are encouraged to add more details about how they plan to implement each engagement activity and the rationale behind their benefit and investment ratings. This information is stored for future reference and may be used when sharing the results of the prioritization tool with colleagues and potential partners.

**Figure 4:**
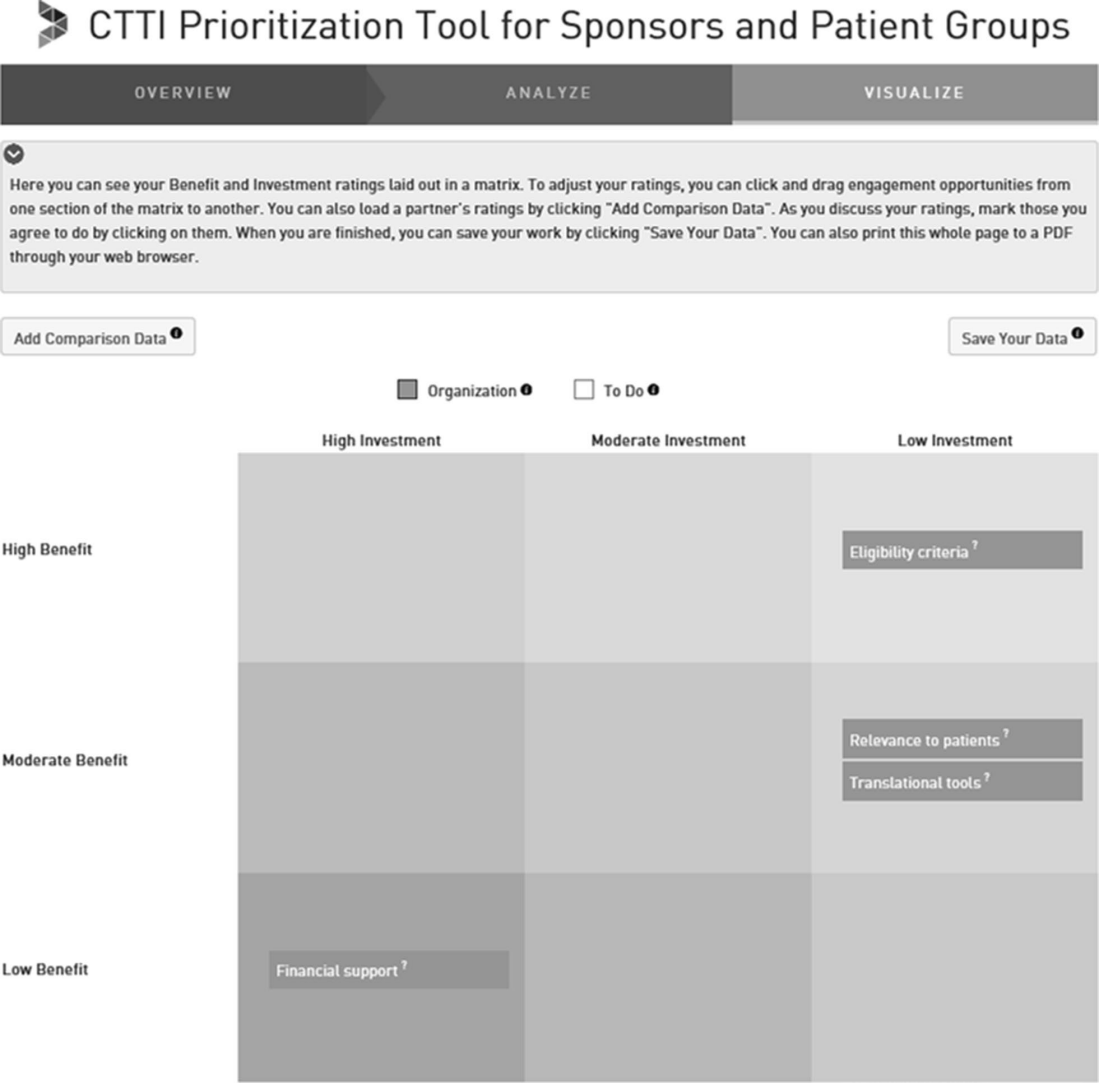
Visualize Patient Engagment Activities Within a Priority Matrix.

After the evaluations are made, the results are visualized in a priority matrix, where each activity is mapped onto a 3 × 3 grid consisting of rows pertaining to the level of benefit the activity is expected to achieve and columns for the level of investment required to perform the activity (Fig. 4). If desired, the user can still adjust its rating (and thus the position of the engagement activity in the matrix) either by going back to the earlier ratings or by placing the particular activity in a different cell in the matrix.

Step three involves identifying mutually beneficial activities. Research sponsors and patient groups interested in working together can compare and discuss priorities to arrive at activities that are of high value for each.

This project and the application of the tool have some limitations. First, we used purposive sampling to select participants who could provide expert experiential knowledge into the various ways patient groups are engaged in medical product development. Their opinions may be different from other patient group and sponsor representatives. Second, this tool does not provide guidance on all factors that influence how or why industry sponsors and patient groups may collaborate in medical product development. There may be a multitude of other activities or benefits or investments that could be considered that are not included in the tool or list of activities. To account for this in the tool, we have provided ways for users to enter their own list of engagement activities. In addition, the benefits and investments listed in the tool are only provided as aspects to consider as the user evaluates the value of the activities. Users are free to evaluate the relative “benefit” or “investment” based on their own understanding of these terms. Third, while revising the list of activities, we were guided by the participants’ feedback on the wording and thoroughness of the list but made our own subjective judgements as to what to revise for the final list. The final list of 24 activities was not re-evaluated using a consensus-seeking process. Future research could explore the breadth and clarity of the final list of engagement activities. Finally, we have not assessed the acceptability or feasibility of the final tool, and have no knowledge of users’ experience with the tool. Future research can be conducted to evaluate users’ willingness to implement the tool, as well as their experience with, and the perceived helpfulness of, the tool when engaging patient groups in medical product development.

## Conclusion

In summary, CTTI has previously developed a foundational set of recommendations for patient group engagement [19, 20]. The recommendations address perceived barriers, including common legal and regulatory concerns, and encourage sponsors, investigators, and other stakeholders to engage with patient groups early and often for better and more efficient clinical trials and to develop meaningful partnerships and demonstrate mutual benefits [19]. The new tool helps implement these recommendations: it allows for up-front and continued collaboration by having both sponsors and patient groups define the level of expected benefit and investment when making decisions on which activities to prioritize. Important next steps may include demonstrating the usefulness of using this tool in fostering meaningful collaborations. Future work could focus on providing example cases where representatives from industry sponsors and patient groups use this tool collectively as a pair to identify and prioritize value-based engagement activities. These case studies may provide useful real-world examples of how the tool can be implemented as well as reveal the impact of intentional industry and patient group partnerships. It is our belief that by examining the comparative value of engagement activities and deciding which activities provide the most benefit for the least investment, meaningful partnerships may be developed that will naturally foster discussions regarding expectations, goals, and specific roles in the design, conduct, and dissemination of research. Future research can evaluate if meaning partnerships do in fact result from using this tool. Ultimately, the impact of meaningful engagement will and should be measured by the resulting usefulness of the information provided by the clinical trial [1].

## Electronic supplementary material

Below is the link to the electronic supplementary material.

Supplementary file1 (DOCX 32 kb)
