# Supplementary material for: Development and Application of a Patient Group Engagement Prioritization Tool for Use in Medical Product Development
Source: Ther Innov Regul Sci. 2020 Sep 29;55(2):324–35. doi: 10.1007/s43441-020-00217-0 (PMC7864807; doi:10.1007/s43441-020-00217-0)
Supplement: Supplementary file 1 — Supplementary file1 (DOCX 32 kb) [file 43441_2020_217_MOESM1_ESM.docx]

**Supplemental material. Description of patient group engagement activities**

| **Engagement activities** | **Description** |
| --- | --- |
| 1. Financial support for research | Patient group contributes directly to a sponsor to support relevant research such as identification of target molecules, characterization of the disease and mechanism of action, or ongoing trial operations. |
| 1. Natural history data | Patient group facilitates the conduct of natural history studies through a registry, access to electronic medical records, aggregation of data, or other methods that aid in understanding the disease. |
| 1. Input on relevance of research to patients | By any of a variety of means-surveys, focus groups, natural language processing of online communities, and tapping existing internal knowledge-the patient group provides data or input to the sponsor on how a planned therapeutic might meet the needs of the patient community. |
| 1. Access to translational tools | Patient group facilitates the development and validation of translational tools such as biomarkers, biosamples, assays, and cell and animal models. |
| 1. Help defining eligibility criteria | Patient group provides feedback based on experience or registry data to determine if the eligibility criteria are practical and appropriate for the patient population. |
| 1. Input on meaningful endpoints & PROs | Patient group provides feedback based on experience, community surveys, focus groups, or registry data on whether the planned clinical endpoints are meaningful and practical for the patient population. |
| 1. Advocacy for policy & funding issues | Patient group communicates between program or review officers and patient group stakeholders; provides education and evidence; and engages in advocacy around policy issues. |
| 1. Education to patient community | Patient group develops audience-appropriate materials such as a lay summary, infographic, or webpage describing the elements of the clinical trial lifecycle. |
| 1. Benefit-risk & patient-preference studies | Patient group facilitates or conducts preference studies to support benefit-risk assessment, or provides feedback from surveys, focus groups, or experience on benefit-risk preferences of the patient population. |
| 1. Protocol design & retention strategy input | Patient group provides input on the protocol and feasibility, and may participate in simulation exercises and suggest improvements that eliminate barriers to participation or minimize burden on patients. |
| 1. Study recruitment & retention strategy input | Patient group provides feedback on recruitment and retention based on geographic, demographic, and symptom profile data in its registry or from experience with prior trials and knowledge of site performance. |
| 1. Increased awareness about trials | Patient group works with sponsor to raise awareness and understanding of the clinical research process. For example, planning an education campaign for a rare disease that does not currently have any trials underway, but will soon. |
| 1. Participant feedback on trial experience | Patient group acts as clearinghouse for feedback from participants on sites and study experience to proactively help manage issues and better plan for future studies. |
| 1. Input on informed consent content & processes | Patient group provides feedback based on experience, community surveys, or focus groups on the content of and processes for providing informed consent. |
| 1. Peer advocates to participants | Patient group staff or volunteer advocate supports trial participants in understanding the informed consent process and acts as an advocate for patients throughout the trial. |
| 1. Clinical trial networks | Patient group creates or supports a clinical network that can be an important resource for research sponsors planning and conducting clinical trials. |
| 1. Data Safety Monitoring Board members | Patient group participates on Data and Safety Monitoring Board to provide perspective on benefit-risk. |
| 1. Support to sponsors around key regulatory meetings | Patient group accompanies sponsor to key meetings with regulators. In a pre-IND meeting, for example, the patient group may provide additional input on issues such as study design, meaningfulness of selected endpoints, and benefit-risk. |
| 1. Support preparing submissions for newborn screening for rare diseases | Patient group assists in the submission of newborn screening for a rare disease if (1) the treatment, if approved, could improve the outcome for patients diagnosed early with the disease; (2) there is sufficient understanding of the disease's natural history, and (3) a newborn screening test for the disease is available and reliable for affected and unaffected infants. |
| 1. Informing regulators on benefit-risk | Patient group provides data and insights to regulators, or performs benefit-risk assessment to inform draft guidance on study design, meaningfulness of selected endpoints, and benefit-risk |
| 1. Public testimony at regulatory meetings | Patient group, while acting as an independent agent, provides defensible data on symptom burden, experience with existing treatments, and benefit-risk preferences at regulatory hearings |
| 1. Support interpreting & disseminating study results | Patient group supports the dissemination of study results in audience-appropriate language and format to relevant stakeholders, such as patients with the same disease/condition, clinicians, and other researchers in the field. |
| 1. Collaboration on post-marketing studies & surveillance initiatives | Patient group may participate in postmarketing study steering committees or working groups that review safety signals, help engage investigators, support data collection via registries, or support recruitment. |
| 1. Support developing access strategy & preparing for value or health technology review | Patient group provides evidence and advocacy in support for coverage or its expansion. |
